# Supplementary material for: Spinal needles versus conventional needles for fine-needle aspiration biopsy of thyroid nodules—A multicenter randomized controlled trial
Source: PLoS One. 2025 Jul 31;20(7):e0321043. doi: 10.1371/journal.pone.0321043 (PMC12312885; doi:10.1371/journal.pone.0321043)
Supplement: S1 File — (DOCX) [file pone.0321043.s001.docx]

# S1: Eligibility Criteria

Inclusion criteria

1. Patients 18 years of age or older.
2. And patients referred for investigation of suspected thyroid cancer with one or more of the following:
   - PET-positive tumor in the thyroid.
   - Or tumor in the thyroid and hoarseness.
   - Or fast-growing tumor in the thyroid.
   - Or hard and immobile tumor in the thyroid.
   - Or tumor in the thyroid with suspect lymph nodes.
3. Or patients referred for evaluation of cold nodules on thyroid scintigraphy and EU-TIRADS $\geq3$.
4. Or patients eligible for radiofrequency ablation treatment

Exclusion criteria

1. FNAB of the same nodule within the last three months.
2. Previously included in the study.
3. Language or other barriers not allowing adequate information.
