# Supplementary material for: Spinal needles versus conventional needles for fine-needle aspiration biopsy of thyroid nodules—A multicenter randomized controlled trial
Source: PLoS One. 2025 Jul 31;20(7):e0321043. doi: 10.1371/journal.pone.0321043 (PMC12312885; doi:10.1371/journal.pone.0321043)
Supplement: S2 File — (DOCX) [file pone.0321043.s002.docx]

# S2: FNAB Technique

# All physicians received instructions followed by supervision according to the following guidelines. Initially, a systematic head and neck ultrasound examination is performed, and a decision is made upon FNAB. The patient's skin is cleaned and disinfected, and a table is prepared with the necessary equipment. The patient is instructed not to move, swallow, or speak during needle insertion. The clinicians can use either ethanol or ultrasound gel on the probe for proper visualization. An in-plane technique visualizes the entire needle path to the nodule. The needle tip should be directed to the solid part of the nodule, which contains minor cystic areas. If the nodule has a sizeable cystic component, the cyst fluid should be drained using a 21G needle before performing the FNAB. According to the physicians' preferences, a syringe without a piston may be attached to the conventional needle for better grip. The needle is held between the thumb and index finger, and the bevel of the needle is oriented towards the transducer. The skin is entered at least 0,5 cm from the edge of the transducer, and when the needle tip reaches the lesion, the needle is moved rapidly back and forth for 3 to 5 seconds with an oscillation frequency of 3 per second. The stylet of the spinal needle is removed once the needle tip is visualized within the lesion of interest for the spinal needle. An assistant may help to remove the stylet. If an assistant is unavailable, the ultrasound probe can be removed once the needle tip is visualized inside the lesion, after which the stylet can be removed using the non-dominant hand. The biopsy is hereafter performed using the capillary technique as with the conventional needle.

The sample material is immediately transferred to the microscope slide for a smear. A 10 ml syringe is attached with the plunger pulled and the sample is expelled near the frosted end of the microscope slide. Another microscope slide is placed above at a 45-degree angle, turned down to a 0-degree angle, and moved perpendicularly to the entire length of the bottom slide in one gentle and quick movement. If a single pass obtains enough sample material, a second droplet is saved for another slide. Three passes are performed on each nodule and the slides are then sent to the regional pathology department. If the initial smear is highly blood-contaminated, the remaining sample is expelled on another microscope slide (hopefully with less blood), and both slides are prepared and sent. If pure cystic material is aspirated on the initial pass, the cyst content is aspirated and sent in a collecting tube before a repeat FNAB.

# Figure S2: FNAB Technique


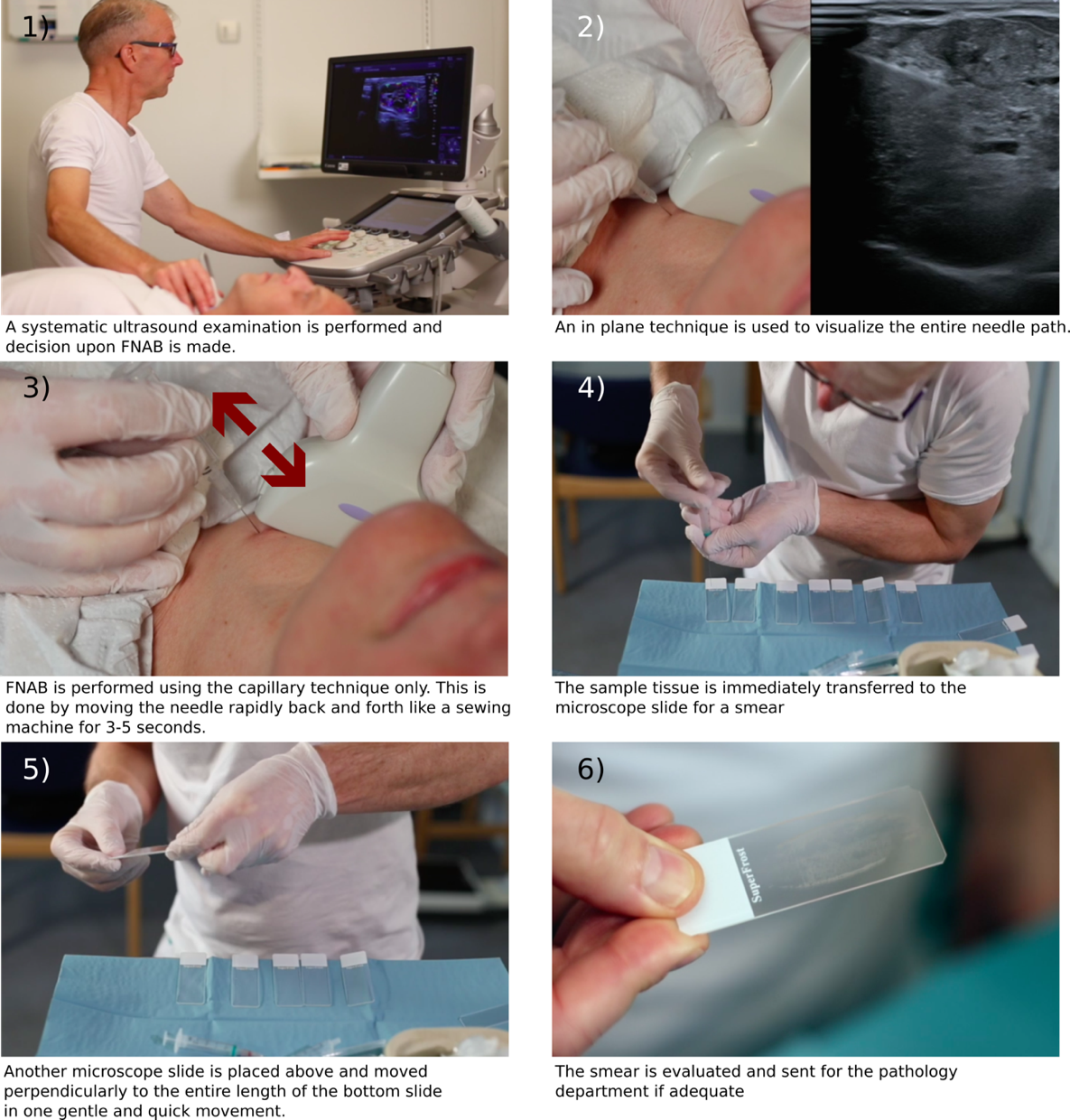


*Abbreviation: FNAB, Fine-Needle Aspiration Biopsy*
