# Supplementary material for: Spinal needles versus conventional needles for fine-needle aspiration biopsy of thyroid nodules—A multicenter randomized controlled trial
Source: PLoS One. 2025 Jul 31;20(7):e0321043. doi: 10.1371/journal.pone.0321043 (PMC12312885; doi:10.1371/journal.pone.0321043)
Supplement: S3 File — (DOCX) [file pone.0321043.s003.docx]

# S3: Sensitivity and Specificity

The cases with cytological diagnoses of suspicious for malignancy (BSRTC category V) or malignancy (BSRTC category VI) that were diagnosed as malignant on final histologic examination were considered true positives. The cases with benign cytology diagnoses (BSRTC category II) that have a surgical specimen with a benign diagnosis were considered to be true negatives. The cases with benign histology that on cytology were classified as suspicious for or with malignancy were classified as false positives. A false-negative diagnosis encompassed cases with malignant histology that were classified as benign on cytology. The cases with the cytological diagnosis of inadequate (BSRTC category I) or follicular neoplasm or suspicious for a follicular neoplasm (BSRTC category IV) were not included in the analysis.

**Table S3A. Cytological and Histological results for the Spinal Group (n=25)**

|  | **Histologically malignant** | **Histologically benign** |
| --- | --- | --- |
| **Cytologically malignant, *suspicious for* malignancy** | 8 | 5 |
| **Cytologically benign** | 1 | 11 |

**Table S3B. Cytological and Histological results for the Control Group (n=17)**

|  | **Histologically malignant** | **Histologically benign** |
| --- | --- | --- |
| **Cytologically malignant, *suspicious for* malignancy** | 6 | 2 |
| **Cytologically benign** | 1 | 8 |
