# Supplementary material for: Spinal needles versus conventional needles for fine-needle aspiration biopsy of thyroid nodules—A multicenter randomized controlled trial
Source: PLoS One. 2025 Jul 31;20(7):e0321043. doi: 10.1371/journal.pone.0321043 (PMC12312885; doi:10.1371/journal.pone.0321043)
Supplement: S4 File — (DOCX) [file pone.0321043.s004.docx]

## S4: Test for Selection Bias

**Table S4.** Gender and age distribution of patients in Zealand University Hospital, who were not included, compared to all the included patients in the trial.

| **Patients** | **N** |  | **Male sex** | **Age** |
| --- | --- | --- | --- | --- |
|  |  |  | N (%) | Mean (SD) |
| Included, all | 359 |  | 91 (25.4%) | 59.7 (14.2) |
| Not included | 517 |  | 126 (24.4%) | 59.9 (14.7) |
| Test of difference  (p-value) |  |  | 0.75 | 0.81 |

Testing the difference in gender distribution was performed using Fisher’s exact test
Testing the difference in age was performed using linear regression
