# Supplementary material for: Spinal needles versus conventional needles for fine-needle aspiration biopsy of thyroid nodules—A multicenter randomized controlled trial
Source: PLoS One. 2025 Jul 31;20(7):e0321043. doi: 10.1371/journal.pone.0321043 (PMC12312885; doi:10.1371/journal.pone.0321043)
Supplement: S5 File — (DOCX) [file pone.0321043.s005.docx]

# S5: Bethesda Categories in the Spinal and Control Groups

**Table S5.** Bethesda Categories in the Spinal and Control Groups

|  | **N** | **Spinal needle** | **Conventional needle** | ***p*-value** |
| --- | --- | --- | --- | --- |
| Bethesda Categories |  |  |  | 0.90 |
| Bethesda Category I | 52 (14.5) | 25 (13.8) | 27 (15.2) |  |
| Bethesda Category II | 249 (69.3) | 127 (70.2) | 122 (68.5) |  |
| Bethesda Category III | 3 (0.8) | 2 (1.1) | 1 (0.6) |  |
| Bethesda Category IV | 30 (8.4) | 13 (7.2) | 17 (9.6) |  |
| Bethesda Category V | 15 (4.2) | 8 (4.4) | 7 (3.9) |  |
| Bethesda Category VI | 10 (2.8) | 6 (3.3) | 4 (2.3) |  |

*Note:* *Values are numbers (%)*
