# Supplementary material for: Spinal needles versus conventional needles for fine-needle aspiration biopsy of thyroid nodules—A multicenter randomized controlled trial
Source: PLoS One. 2025 Jul 31;20(7):e0321043. doi: 10.1371/journal.pone.0321043 (PMC12312885; doi:10.1371/journal.pone.0321043)
Supplement: S6 File — (DOCX) [file pone.0321043.s006.docx]

## S6: Expertise Level at Inclusion Sites

**Table S6.** The number of different doctors and levels of expertise at each inclusion site

| Experience level | **Zealand University Hospital** | **Rigshospitalet** | **Herlev University Hospital** |
| --- | --- | --- | --- |
| $>$4 years of experience | 6 (31.6) | 7 (53.8) | 3 (100) |
| $\leq$4 years of experience | 13 (68.4) | 6 (46.2) | - |
| **Total** | 19 | 13 | 3 |

*Note:* *Values are numbers (%)*
