# Supplementary material for: Spinal needles versus conventional needles for fine-needle aspiration biopsy of thyroid nodules—A multicenter randomized controlled trial
Source: PLoS One. 2025 Jul 31;20(7):e0321043. doi: 10.1371/journal.pone.0321043 (PMC12312885; doi:10.1371/journal.pone.0321043)
Supplement: S8 File — (DOCX) [file pone.0321043.s008.docx]

# S8: Codebook

| **#** | **Variable / Field Name** | **Field Label**  ***Field Note*** | **Field Attributes (Field Type, Validation, Choices, Calculations, etc.)** |
| --- | --- | --- | --- |
| **Instrument:SPINAB(my_first_instrument) Collapse** | | | |
| 1 | [record_id] | Record ID | text |
| 9 | [Nr] | Participant numbe | text, Required, Identifier Custom alignment: RH |
| 10 | [Hospital] | Hospital | radio   \| 1 \| Herlev \| \| --- \| --- \| \| 2 \| Rigshospitalet \| \| 3 \| Køge \| |
| 11 | [Randomization] | Randomization | radio   \| 1 \| Intervention (spinal needle) \| \| --- \| --- \| \| 2 \| Control (control needle) \|   Custom alignment: RH |
| 13 | [Gender] | Gender | radio   \| 0 \| Female \| \| --- \| --- \| \| 1 \| Male \|   Custom alignment: RH |
| 14 | [Age] | Age | text |
| 15 | [Inclusions critiera] | Inclusion criteria | radio   \| 1 \| Cold nodule on scintigraphy and EU-TIRADS 3 or higher \| \| --- \| --- \| \| 2 \| Reasonable suspicion of cancer \| |
| 16 | [eutirads] | EU-TIRADS | radio   \| 1 \| Score 3 (and >/= 20 mm in size) \| \| --- \| --- \| \| 2 \| Score 4 (and >/= 15 mm in size)) \| \| 3 \| Score 5 (and >/= 10 mm in size)) \| |
| 17 | [cancer]  Show the field ONLY if:  [inkl_krit] = '2' | Begrundet mistanke for kræft  *Angiv én eller flere* | checkbox   \| 1 \| inkl_krit_cancer___1 \| PET positive nodule \| \| --- \| --- \| --- \| \| 2 \| inkl_krit_cancer___2 \| Thyroid nodule AND suspected recurrent laryngeal palsy \| \| 3 \| inkl_krit_cancer___3 \| Fast growing nodule \| \| 4 \| inkl_krit_cancer___4 \| Hard and immobile nodule \| \| 5 \| inkl_krit_cancer___5 \| Thyroid nodule and suspicious lymph nodes \| |
| 18 |  |  |  |
| 19 |  |  |  |
| 20 | [Background] | Baggrund  *Angiv en eller flere* | checkbox   \| 1 \| baggrund___1 \| Family history of thyroid cancer \| \| --- \| --- \| --- \| \| 2 \| baggrund___2 \| Radiation on the neck \| \| 3 \| baggrund___3 \| Previous thyroid surgery \| \| 4 \| baggrund___4 \| Previous neck surgery \| \| 5 \| baggrund___5 \| Metabolic disease \| \| 6 \| baggrund___6 \| Previous thyroid cancer \| \| 7 \| baggrund___7 \| Previous neck cancer \| \| 8 \| baggrund___8 \| Previous cancer \| \| 9 \| baggrund___9 \| Previous endocrine neoplasia \| \| 10 \| baggrund___10 \| Not to all above \| \| 99 \| baggrund___99 \| Not specified \| |
| 21 | [radioiodine] |  | radio   \| 1 \| Yes \| \| --- \| --- \| \| 2 \| No \| \| 3 \| Not specified \| |
| 22 | [blood_thinner] |  | radio   \| 1 \| Yes \| \| --- \| --- \| \| 2 \| No \| \| 99 \| Not specified \| |
| 23 | [ak_med_1]  Show the field ONLY if:  [ak_med] = '1' | Præparat  *Angiv én eller flere* | checkbox   \| 1 \| ak_med_1___1 \| Vitamin K-Antagonister: Kumariner (Warfarin (Marevan®) og Phenprocoumon (Marcoumar®) \| \| --- \| --- \| --- \| \| 2 \| ak_med_1___2 \| Fondaparinuxnatrium (Arixtra®) \| \| 3 \| ak_med_1___3 \| Lavmolekylært heparin (Low Molecular Weight Heparin (LMWH)), f.eks.: Dalteparin (Fragmin®), Tinzaparin (Innohep®), Enoxaparin (Klexane®) \| \| 4 \| ak_med_1___4 \| Dabigatran etexilate (Pradaxa®) \| \| 5 \| ak_med_1___5 \| Apixaban (Eliquis®) \| \| 6 \| ak_med_1___6 \| Rivaroxaban (Xarelto®) \| \| 7 \| ak_med_1___7 \| Edoxaban (Lixiana®) \| \| 8 \| ak_med_1___8 \| Dipyridamol (Persantin®) \| \| 9 \| ak_med_1___9 \| ASA/Magnyl \| \| 10 \| ak_med_1___10 \| Clopidogrel (Plavix®) \| \| 11 \| ak_med_1___11 \| Ticagrelor (Brilique®) \| \| 12 \| ak_med_1___12 \| Prasugrel (Efient®) \| |
| 24 | [dosis]  Show the field ONLY if:  [ak_med] = '1' | dosis  *mg eller IE* | text |
| 25 | [ak_med_frequency]  Show the field ONLY if:  [ak_med] = '1' |  | text |
| 26 | [smoking] | Smoking | radio   \| 1 \| Nihil \| \| --- \| --- \| \| 2 \| Previous smoker \| \| 3 \| Smoker \| \| 99 \| Not specified \| |
| 28 | [alcohol] | Alcohol | radio   \| 1 \| No \| \| --- \| --- \| \| 2 \| Yes \| |
| 31 | [obj_andet]  Show the field ONLY if:  [obj(5)] = '1' | Andet | text |
| 32 | [obj_karakter]  Show the field ONLY if:  [obj(1)] = '1' | Characteristics of palpation | checkbox   \| 1 \| obj_karakter___1 \| Hard \| \| --- \| --- \| --- \| \| 2 \| obj_karakter___2 \| Soft \| \| 3 \| obj_karakter___3 \| Mobile \| \| 4 \| obj_karakter___4 \| Fixed \| \| 99 \| obj_karakter___99 \| Not specified \| |
| 35 | [tsh]  Show the field ONLY if:  [biokemi]='1' | TSH  *x 10^-3 int.enh./L (kun tal)* | text |
| 36 | [Eutirads_us] | Section Header: *ULTRASOUND*  EU-TIRADS | radio   \| 1 \| 2 \| \| --- \| --- \| \| 2 \| 3 \| \| 3 \| 4 \| \| 4 \| 5 \| \| 9 \| Ej angivet \| |
| 37 | [us_biggest] | *mm* | text |
| 38 | [ul_depth] | *mm* | text |
| 39 | [ul_height] | *mm* | text |
| 40 | [US_scucture] |  | radio   \| 1 \| Cystic \| \| --- \| --- \| \| 2 \| Cystic-solid \| \| 3 \| Solid \| \| 4 \| Spongioform \| \| 5 \| Not specified \| |
| 41 | [ul_highrisk]  Show the field ONLY if:  [ul_eutirads] = '4' |  | checkbox   \| 1 \| ul_highrisk___1 \| Not ovale shape \| \| --- \| --- \| --- \| \| 2 \| ul_highrisk___2 \| Irregular \| \| 3 \| ul_highrisk___3 \| Microcalcifikations \| \| 4 \| ul_highrisk___4 \| HYPOekkoisk \| |
| 42 | [ul_doppler] | Doppleraktivitet af tyroideaknuden | radio   \| 1 \| Avascular \| \| --- \| --- \| \| 2 \| Normovascular \| \| 3 \| Increased edge flow \| \| 4 \| Increased intramodular flow \| \| 99 \| Not specified \| |
| 43 | [Lymf] | Pathological lymph nodes | radio   \| 1 \| No \| \| --- \| --- \| \| 2 \| Yes \| \| 3 \| Not specified \| |
| 48 | [Cyst_fluid] | Cyst fluid removed | yesno   \| 1 \| Yes \| \| --- \| --- \| \| 0 \| No \| |
| 50 | [Complications] |  | checkbox   \| 1 \| komplikation___1 \| Ingen beskrevet \| \| --- \| --- \| --- \| \| 2 \| komplikation___2 \| Blødning/hæmatom (Indlæggelses- eller behandlingskrævende) \| \| 3 \| komplikation___3 \| Infektion (behandlingskrævende) \| \| 4 \| komplikation___4 \| Recurrensparese \| \| 5 \| komplikation___5 \| Horners syndrom \| \| 6 \| komplikation___6 \| Anden \| |
| 52 | [NRS] | Pain | dropdown   \| 1 \| 0 \| \| --- \| --- \| \| 2 \| 1 \| \| 3 \| 2 \| \| 4 \| 3 \| \| 5 \| 4 \| \| 6 \| 5 \| \| 7 \| 6 \| \| 8 \| 7 \| \| 9 \| 8 \| \| 10 \| 9 \| \| 11 \| 10 \| |
| 53 | [Patolog_1] | Section Header: *Patologisvar j.f. Bethesda - Se afkrydsningsskemaet. BEMÆRK her menes ikke diagnosen til klinikerne.*  Patolog 1  *Angiv initialer. MR: Marie Rosenørn, KK: Katalin Kiss, GL: Giedrius Lelkeitis, LA: Louise Andersen* | text |
| 54 | [Patolog_2] | Patolog 2  *Angiv initialer. MR: Marie Rosenørn, KK: Katalin Kiss, GL: Giedrius Lelkeitis, LA: Louise Andersen* | text |
| 55 | [Diagnostic_adeqaute] |  | radio   \| 1 \| Non-diagnostic \| \| --- \| --- \| \| 2 \| Diagnostic \| |
| 56 | [nondiagnostic_cause]  Show the field ONLY if:  [diagnostisk] = '1' | Cause | checkbox   \| 1 \| nondiagnostic_cause___1 \| Cyst fluid only: PYYBT0 \| \| --- \| --- \| --- \| \| 2 \| nondiagnostic_cause___2 \| Virtually acellular specimen: M0901Y \| \| 3 \| nondiagnostic_cause___3 \| Obscuring blood: M09015 \| \| 4 \| nondiagnostic_cause___4 \| Other: clotting artifact, etc: M09030 \| \| 5 \| nondiagnostic_cause___5 \| Too few cells (< 60): M0901X, M09000 \| |
| 57 | [nondiagnostisk_other]  Show the field ONLY if:  [nondiagnostic_cause(4)] = '1' | Udspecificer årsag til non-diagnostisk | text |
| 58 | [Diagnosis]  Show the field ONLY if:  [diagnostisk] = '2' | Udspecificer diagnose | radio   \| 1 \| II. BENIGN \| \| --- \| --- \| \| 2 \| III. ATYPIA OF UNDETERMINED SIGNIFICANCE or FOLLICULAR LESION OF UNDETERMINED SIGNIFICANCE \| \| 3 \| IV. FOLLICULAR NEOPLASM or SUSPICIOUS FOR A FOLLICULAR NEOPLASM \| \| 4 \| V. SUSPICIOUS FOR MALIGNANCY \| \| 5 \| VI. MALIGNANT \| |
| 59 | [benign]  Show the field ONLY if:  [diagnostic_cause] = '1' |  | radio   \| 1 \| Consistent with a benign follicular nodule (includes adenomatoid nodule, colloid nodule, etc.) \| \| --- \| --- \| \| 2 \| Consistent with lymphocytic (Hashimoto) thyroiditis in the proper clinical context \| \| 3 \| Consistent with granulomatous (subacute) thyroiditis \| \| 4 \| Other \| |
| 61 | [diagnostic_neoplasm]  Show the field ONLY if:  [diagnostic_cause] = '3' | Specify if Hürthle cell (oncocytic) type | yesno   \| 1 \| Yes \| \| --- \| --- \| \| 0 \| No \| |
| 62 | [diagnostic_suspicious]  Show the field ONLY if:  [diagnostic_cause] = '4' | Udspecificer diagnosen | radio   \| 1 \| Suspicious for papillary carcinoma \| \| --- \| --- \| \| 2 \| Suspicious for medullary carcinoma \| \| 3 \| Suspicious for metastatic carcinoma \| \| 4 \| Suspicious for lymphoma \| \| 5 \| Other \| |
| 63 | [diagnostic_suspicious2]  Show the field ONLY if:  [diagnostic_suspicious] = '5' | Udspecificer "Other" | text |
| 64 | [diagnostic_malignant]  Show the field ONLY if:  [diagnostic_cause] = '5' | Udspecificer diagnosen | radio   \| 1 \| Papillary thyroid carcinoma \| \| --- \| --- \| \| 2 \| Poorly differentiated carcinoma \| \| 3 \| Medullary thyroid carcinoma \| \| 4 \| Undifferentiated (anaplastic) carcinoma \| \| 5 \| Squamous-cell carcinoma \| \| 6 \| Carcinoma with mixed features (specify) \| \| 7 \| Metastatic carcinoma \| \| 8 \| Non-Hodgkin lymphoma \| \| 9 \| Other \| |
| 66 | [cytologi_danish] | Section Header: *Patologisvar - Efter dansk definition (se patoweb)*  Patologisvar til klinikere (dvs. j.f. dansk definition). Se patoweb for denne. | radio   \| 1 \| Non-diagnostic \| \| --- \| --- \| \| 2 \| Diagnostic \| |
| 67 | [nd_dansk]  Show the field ONLY if:  [cytologi_danish] = '1' | Årsag non-diagnostisk  *Vælg en eller flere* | checkbox   \| 1 \| nd_dansk___1 \| Cyst fluid only: PYYBT0 \| \| --- \| --- \| --- \| \| 2 \| nd_dansk___2 \| Virtually acellular specimen: M0901Y \| \| 3 \| nd_dansk___3 \| Obscuring blood: M09015 \| \| 4 \| nd_dansk___4 \| Other: clotting artifact, etc: M09030 \| \| 5 \| nd_dansk___5 \| Too few cells (< 60): M0901X, M09000 \| \| 6 \| nd_dansk___6 \| Too few cells (60-120 celler): M09000 \| \| 7 \| nd_dansk___7 \| Colloid, no cells: PYYCOL \| |
| 68 | [histology] | Section Header: *Histologi*  Foreligger histologi? | yesno   \| 1 \| Yes \| \| --- \| --- \| \| 0 \| No \| |
| 69 | [histology_surgery]  Show the field ONLY if:  [histologi] = '1' | Histologisvar | radio   \| 1 \| Malignt \| \| --- \| --- \| \| 2 \| Follikulært adenom \| \| 3 \| Benign \| |
