# Supplementary material for: Spinal needles versus conventional needles for fine-needle aspiration biopsy of thyroid nodules—A multicenter randomized controlled trial
Source: PLoS One. 2025 Jul 31;20(7):e0321043. doi: 10.1371/journal.pone.0321043 (PMC12312885; doi:10.1371/journal.pone.0321043)
Supplement: Attachment — Submitted filename: Response to Reviewers.docx [file pone.0321043.s011.docx]

Dear Editor,

We thank you and the reviewers for your time and the insightful comments on our manuscript, “Spinal Needles Versus Conventional Needles for Fine-Needle Aspiration Biopsy of Thyroid Nodules – A Multicenter Randomized Controlled Trial.”
We have carefully considered all the feedback and revised the manuscript accordingly. Below we provide a point-by-point response to each comment (with the responses quoted in red text for clarity). All changes to the manuscript are clearly highlighted in the revised version. We believe these revisions address the concerns raised and have improved the manuscript. We are grateful for the opportunity to clarify our work. Thank you again for your constructive input.

**Reviewer #1 Comments:**

**Reviewer 1 – Comment 1:** ”A logistic regression model was used to analyze the primary outcome with respect to the two types of needles. In accordance to the ICH E9 guideline “Statistical principles for clinical trials”, the main treatment effect should be investigated using a statistical model that allows for centre differences. I.e. the centre effect should be included in the statistical model of the primary analysis. Subsequently, the treatment by centre interaction should be evaluated in a sensitivity analysis and carefully discussed.”

**Response 1:** We appreciate this important comment. We agree that accounting for the multicenter design is essential. In fact, we did account for center effects in our analysis, but we recognize that our original description was not sufficiently explicit. To address this, we have modified the Statistical Analysis section to emphasize that we used a clustered modeling approach.

Specifically, we now write: *“To adjust for the multicenter design, analyses of the primary outcome were conducted using a generalized estimating equation (GEE) logistic regression model with study center as a clustering variable. Similarly, clustering by operator was considered to account for any physician-related effects.”* (Page 10, lines 213-217)

We also note in the results that this approach did not materially change the findings (the primary outcome results remained essentially the same after adjusting for center). By clarifying our use of a GEE model (with centers as clusters), we have addressed the reviewer’s concern. If needed, we are prepared to provide further details or additional analyses regarding center effects. We hope this explanation satisfies the reviewer.

**Reviewer 1 – Comment 2:** The significance level for all analyses was set to 5%. Considerations how multiplicity of analyses will be taken into account (e.g. appropriate adjustment of the significance level) should be provided.

**Response 2:** Thank you for highlighting the need to explain our strategy for type-I-error control. We prespecified **one single primary endpoint**, the proportion of adequate cytological samples and powered the trial exclusively for that comparison. Consequently, the family-wise error rate for efficacy is fully controlled at α = 0.05 for the primary analysis, and no further adjustment is required for that single test (ICH E9 §2.2.2).

All **secondary** (pain score, complications, diagnostic accuracy) **and subgroup analyses** were explicitly labelled exploratory in the original protocol and statistical-analysis plan. In line with CONSORT and ICH guidance, we therefore report these results with nominal two-sided p-values and 95 % confidence intervals for descriptive insight only, without formal multiplicity correction. To enhance transparency we have now added the following to the method and discussion:

*“The overall trial alpha was fixed at 0.05 for the single, prespecified primary outcome. Secondary and subgroup analyses were exploratory; accordingly, their p-values are presented without multiplicity adjustment and any findings should be regarded as hypothesis-generating.” (Page 10, lines 233-236))*

*“Furthermore, because only one primary comparison was powered and multiplicity adjustment was not applied to exploratory analyses, positive signals among secondary outcomes should be interpreted with caution.” (Page 18, lines 400-402)*

**Reviewer 1 – Comment 3:** Sample size calculation is one of the most important steps in the design of a clinical trial and should be presented in a transparent and understandable manner. The number of patients required has been calculated based on an expected improvement of 10% in the primary outcome, resulting in 175 patients in each group with a significance level of 5% and a power of 80%. This information is not sufficient for a clear understanding of the calculation. For example, if a chi-square test is used based on a 10% change from 75% to 85%, approximately 248 patients are needed, but for a change from 86% to 96%, 125 patients are needed. The number will be different for another statistical model, such as a stratified logistic regression model. Therefore, the used statistical model as well as the basic rate in addition to the expected change is a necessary piece of Information.

**Response 3:** We thank Reviewer #1 for pointing out the need for clarification on the sample size calculation. We have revised the Methods section to clearly explain our a priori power analysis and assumptions. In the revised manuscript, we now state:

*“The sample size was determined based on an expected 10% absolute increase in adequate cytological diagnoses (from 86% in the control group to 96% in the spinal needle group, as suggested by prior studies (16,18). Using a two-sided α of 0.05 and power (1–β) of 90%, we calculated that 175 patients per group (350 total) would be required to detect this difference (19).”* (Page 9, lines 197-201)

This added text details the effect size assumption, significance level, and power that underpinned our sample size calculation. We trust that this clarification addresses the reviewer’s concern.

**Reviewer 1 – Comment 4:** “It would also be helpful to explain why 380 patients were randomised although only 350 were to be included in the study.” In addition, contradictory data regarding the analyzed numbers of patients can be found in the flow-diagram (Figure 1, Analysed spinal needle 178, conventional needle 181) and in Table 2 (spinal needle 181, conventional needle 178).

**Response 4:**

We agree that clarification is warranted. **Our target was to analyze 350 participants who actually underwent the allocated FNAB.** To reach this figure we continued randomizing until 350 procedures had been completed, anticipating that a small proportion of randomized patients might not proceed to biopsy. In practice, 380 envelopes were opened, but 21 patients did not receive the intervention and therefore contributed no data. The most common reasons were:

- withdrawal of consent immediately after randomization (n = 9),
- clinical decision not to perform FNAB (n = 6)
- envelope opened in error for an ineligible patient (n = 6).

Thus, 359 participants received the intervention thereby exceeding the prespecified information size.

We therefore added following:

“*The calculated sample size (n = 350) referred to participants who actually underwent the allocated FNAB. To compensate for anticipated post- randomization drop-outs (~10 %), we continued enrolment until ≥ 350 procedures had been completed. Enrollment stopped after 380 envelopes had been opened; 21 patients did not proceed to, leaving 359 analyzable participants.”* (Page 9, lines 202-206)

We trust that this focused explanation resolves the reviewer’s concern.

Thank you for drawing our attention to this inconsistency. The numbers in **Table 2 are correct** (spinal needle = 181; conventional needle = 178). The values were inadvertently transposed in the final box of the CONSORT flow diagram.

- We have replaced Figure 1 with a corrected version that now reads
  **“Analyzed: spinal needle (n = 181); conventional needle (n = 178).”**

**Reviewer #2 Comments:**

**Reviewer 2 – Comment:** The authors presented a very interesting article concerning the current topic regarding the choice of thyroid biopsy needle. Considering the large number of patients who undergo this procedure all over the world, the choice of needle - standard or spinal, that is, the result obtained by using one or the other, as well as the accompanying side effects and complications, is of great importance. Earlier studies showed the advantage of the spinal needle, however, this study shows that there is no such advantage, and when one takes into account the significantly higher price of the spinal needle compared to the standard needle, a clear conclusion is reached that the current practice of using the standard needle is satisfactory. The study was well designed and conducted and clearly presented. The conclusions are plain and applicable in everyday work. I am of the opinion that in the text of the article it is not necessary to state the exact names of the institutions and persons who performed any procedures, and that is the only correction that needs to be made.

**Response:** We thank the reviewer for the positive assessment of our work and for the helpful suggestion regarding textual economy.

**Institutions:** In the Methods section (“Study design and population”) we originally listed the full names of the three recruiting departments to document the multicentre setting. As this information may still be useful to readers evaluating external validity, we have kept the hospital names but streamlined the wording (e.g. “Department of Otorhinolaryngologyt” rather than the full official title and place).

**Individuals:** We have removed the names of individual physicians and technicians from the narrative text, retaining only those required in the author list and the brief acknowledgment of Ms Tina K. Agander for cytological slide preparation (as permitted by PLOS ONE). No procedural details now identify specific persons.

These edits shorten the manuscript and preserve confidentiality without compromising methodological transparency. We appreciate the reviewer’s thoughtful remark.

**Reviewer #3 Comments:**

**Reviewer 3 – Comment 1:** ”While the randomization was centralized and pre-generated, it is not clear how allocation was concealed (e.g., via sealed opaque envelopes, secure web-based tool, third-party randomization center). Authors to explicitly describe the allocation concealment mechanism. This is critical to avoid selection bias. Did not mention operator blinding or cytopathologist blinding, which should be clarified ”

**Response 1:** We appreciate this important observation. Allocation was rigorously concealed by using the **sequentially-numbered, opaque, sealed-envelope**, prepared and stored by personnel who had **no role in patient enrollment**. The two authors (TVA and KDL) created the center-stratified, block- randomization list (block size = 10) with [www.randomization.com](http://www.randomization.com). A research secretary not involved in recruitment transferred each code to a tamper-evident, opaque envelope, numbered in chronological order. Each envelope contained: (i) the assignment (“spinal” or “conventional”), (ii) a colour-coded needle sticker so the operator could immediately select the correct device, and (iii) the participant ID label. The clinician opened the next envelope **in sequence** before the biopsy; thus neither investigators nor participants could foresee the assignment. Cytopathologists and data analysts remained blinded to group allocation throughout the study.

**Randomization and Allocation Concealment:**
We now explain that:
*“Participants were randomly assigned (1:1) to the spinal needle group or the conventional needle group using a computer-generated random sequence with permuted block sizes (=10). The random sequence was generated by two investigators before the start of the trial* using the website [*www.randomization.com*](http://www.randomization.com)*. To ensure strict allocation concealment, the group assignments were sealed in sequentially numbered, opaque envelopes prepared before the start of the trial. Each envelope contained the designated needle (spinal or conventional) and was only opened by the clinician after the patient had been enrolled.”* (Page 8, lines 174-182)

This clarification should reassure the reviewer that the randomization process was properly concealed and prevented any potential selection bias.

**Blinding:**
We have also clarified the blinding in our trial. The revised text reads:

*“This trial was single-blinded. The patients were not informed about needle type used for their biopsy, and importantly, the cytopathologists were blinded as for the needle type. The clinicians performing the FNAB procedure, however, could not be blinded due to the obvious differences in needle appearance and handling, these operators were aware of the needle type upon opening the envelope. No other personnel had access to allocation information during the trial.”* (Page 9, lines 188-194)

**Reviewer 3 – Comment 2: “**Was any subgroup analysis performed for deep vs superficial nodules? This would greatly increase the clinical value.”

**Response 2:** We appreciate the suggestion and agree that this could affect the diagnostic rate. However, **no subgroup analysis by nodule depth was performed** for the following reason: Depth was not systematically recorded for every participant at any of the three centers. Even if this were information available, the study was powered solely for the primary endpoint in the overall cohort, not for additional depth-stratified comparisons.

**Reviewer 3 – Comment 3:** “Limitations section should be expanded:

1. Subjective pain measurement.
2. Operator variability (e.g., junior vs senior radiologist).
3. Blinding limitations.”

**Response 3:** Thank you for this relevant comment. We have now added this to the limitations in the Discussion:
*“This study has several limitations that warrant consideration.* ***First****, pain was assessed with a single, patient-reported 0–10 Numerical Rating Scale immediately after biopsy. Such subjective measurement is susceptible to individual pain-threshold variation.* ***Second****, although we adjusted for operator experience (> 4 years vs ≤ 4 years) in the multivariable models, other aspects of operator variability (e.g. specialty background, annual biopsy volume, junior vs senior status) could not be fully captured and may still influence cytological adequacy.* ***Third****, blinding was incomplete, patients and cytopathologists were blinded to needle type, but the performing clinicians could not be blinded because of obvious visual and tactile differences between the needles, introducing a potential performance bias.* ***Fourth****, A limitation of our study is that only 26.1% of patients referred for thyroid FNAB at the participating hospitals were enrolled in the study. This is mainly due to the SPINAB trial being conducted in a real-world clinical setting, where limited time and availability did not allow all patients to be invited. Further, only physicians who received instructions and hands-on training in the use of spinal needle FNAB could include patients. We prioritized the training of physicians in the spinal needle technique to ensure standardized performance in the spinal group. Consequently, we could not include all patients referred for FNAB. However, since our post hoc analysis did not find any differences between the included vs. non-included patients in the SPINAB trial, we believe the results are generalizable beyond the included patients (S4).”* (Page 16-17, line 350-370)

**Reviewer 3 – Comment 4:** “Discussion should cite other RCTs (if any exist) or similar cohort studies and explain how this study adds new evidence.”

**Response 4:** To our knowledge, no previous randomized controlled trial has compared spinal versus conventional needles for thyroid FNAB. We have therefore reinforced the Discussion by contrasting our findings with the three largest prior non-randomized studies and explaining the incremental value of our RCT design:

“*Prior to the current study, three non-randomized prospective studies by Cappelli et al. and Ahari et al. have been performed, including 516, 320, and 240 patients, respectively (16-18). These studies found that spinal needles reduce inadequate samples by 6–12 percentage points(16–18). However, those studies were single-center, relied on a single operator and lacked randomization, raising the possibility of selection and performance biases.* ***Our multicenter RCT, involving 23 operators across three hospitals, provides the first high-level evidence that spinal needles do not confer a clinically relevant advantage when rigorous randomization and blinding of outcome assessment are applied*.**” (Page 17-18, lines 372-380)

**Editorial Comments (PLOS ONE Editorial Office):**

**Editorial Comment 1:** *“Please ensure that your manuscript includes all required statements per PLOS ONE guidelines. In particular, add or update the following: (a) a Funding Disclosure, (b) a Competing Interests (Conflict of Interest) statement, and (c) a Data Availability Statement that clearly describes where and how the data can be accessed. In addition, if your submission contains any images or identifiable information from study participants, please confirm that you have obtained informed consent for publication of these from the individuals (or state if not applicable).”*

**Response to Comment 1:** We appreciate the reminder to include these important statements. We have updated the manuscript as requested:

- **Funding Statement:** We have now removed the Funding Disclosure section from the article as suggested and added it to the cover letter. It now reads: *“Funding****:*** *This work was supported by the Erik and Susanna Olesen Fund (grant no. #). The funder had no role in study design, data collection and analysis, decision to publish, or preparation of the manuscript.”*
- **Competing Interests:** We have now removed the Competing Interests statement in the manuscript. At add the following to the cover letter: *“Competing Interests: The authors have declared that no competing interests exist.”*.
- **Image Consent Compliance:** We confirm that our manuscript does not contain any identifiable images of patients or participants. The two individuals depicted in the images in Figure S2 are co-authors involved in the trial. In the Methods sections, we have stated: “*Informed consent was given by the two co-authors depicted in the images of Figure S2*”. Additionally, all participants in our study provided written informed consent for participation (including consent for use of anonymized data and images). This should ensure full compliance with PLOS ONE’s image and ethics requirements.

**Editorial Comment 2:** When completing the data availability statement of the submission form, you indicated that you will make your data available on acceptance. We strongly recommend all authors decide on a data sharing plan before acceptance, as the process can be lengthy and hold up publication timelines. Please note that, though access restrictions are acceptable now, your entire data will need to be made freely accessible if your manuscript is accepted for publication. This policy applies to all data except where public deposition would breach compliance with the protocol approved by your research ethics board. If you are unable to adhere to our open data policy, please kindly revise your statement to explain your reasoning and we will seek the editor's input on an exemption. Please be assured that, once you have provided your new statement, the assessment of your exemption will not hold up the peer review process.

**Response to Comment 2:** We fully endorse PLOS ONE’s data-sharing policy. A completely de-identified, participant-level dataset together with detailed codebook have been uploaded as Supporting Information files accompanying this submission. We believe this resolves the data-availability concern.

**Revised Data Availability Statement (to be inserted in the manuscript)**

*All de-identified individual-participant data that underpin the results in this article, the final trial protocol, and a full data dictionary/codebook are provided as Supporting Information files: Dataset (“SPINAB_trial_dataset.xlsx”), Codebook (“Codebook.pdf”). No additional restrictions apply. (Page 21, Line 472-476)*

We have addressed all the points raised by the reviewers and the editorial office, and we have revised the manuscript accordingly. Once again, we thank the reviewers and editors for their thorough review and constructive feedback, which have helped us improve the clarity and quality of our paper. We hope that our responses and the revised manuscript meet all the requirements and concerns.

Sincerely,
*Kasper Daugaard Larsen*
On behalf of all authors of the manuscript
